# F-actin bending facilitates net actomyosin contraction By inhibiting expansion with plus-end-located myosin motors

**DOI:** 10.1007/s00285-022-01737-z

**Published:** 2022-07-04

**Authors:** Alexander K. Y. Tam, Alex Mogilner, Dietmar B. Oelz

**Affiliations:** 1 UniSA STEM, The University of South Australia, Mawson Lakes Campus, Mawson Lakes, SA 5095 Australia; 2 School of Mathematics and Physics, The University of Queensland, St Lucia Campus, St Lucia, 4072 Queensland Australia; 3 Courant Institute of Mathematical Sciences, New York University, New York, 10012–1185 NY USA

**Keywords:** Actomyosin, Curve-straightening flow, Energy functional, Gradient flow, Stress tensor, Asymptotic analysis, 35A01, 65L10, 65L12, 65L20, 65L70

## Abstract

Contraction of actomyosin networks underpins important cellular processes including motility and division. The mechanical origin of actomyosin contraction is not fully-understood. We investigate whether contraction arises on the scale of individual filaments, without needing to invoke network-scale interactions. We derive discrete force-balance and continuum partial differential equations for two symmetric, semi-flexible actin filaments with an attached myosin motor. Assuming the system exists within a homogeneous background material, our method enables computation of the stress tensor, providing a measure of contractility. After deriving the model, we use a combination of asymptotic analysis and numerical solutions to show how F-actin bending facilitates contraction on the scale of two filaments. Rigid filaments exhibit polarity-reversal symmetry as the motor travels from the minus to plus-ends, such that contractile and expansive components cancel. Filament bending induces a geometric asymmetry that brings the filaments closer to parallel as a myosin motor approaches their plus-ends, decreasing the effective spring force opposing motor motion. The reduced spring force enables the motor to move faster close to filament plus-ends, which reduces expansive stress and gives rise to net contraction. Bending-induced geometric asymmetry provides both new understanding of actomyosin contraction mechanics, and a hypothesis that can be tested in experiments.

## Introduction

The mechanics of actin filaments and myosin motor proteins in the cell cortex underpins movement (Yamada and Sixt 2019) and division (Pollard 2010) of biological cells. Early breakthroughs in understanding actomyosin dynamics occurred in muscle cells (Gautel 2011), in which actin and myosin form sarcomere structures. Sarcomeres involve filaments aligned in parallel with minus-ends in the centre and plus-ends pointing outwards. Relative motion of myosin motors towards filament plus-ends subsequently generates contraction by pulling filaments inwards. This mechanism is known as sliding filament theory (Huxley 2004). However, actomyosin networks in the cell cortex are disordered, with filaments distributed at random. Experiments (Murrell et al. 2015; Pollard and O’Shaughnessy 2019) and simulations (Tam et al. 2021; Ennomani et al. 2016) have shown that disordered actomyosin networks also contract (Chalut and Paluch 2016). According to sliding filament theory, filament pairs produce expansion if myosin motor proteins localise close to plus-ends, or contraction if myosin motor proteins localise close to minus-ends. In disordered actomyosin networks, motors localise near plus-ends and minus-ends with equal probability. Therefore, sliding filament theory alone cannot explain disordered network contraction. The origin of contraction in disordered actomyosin networks remains an active field of research.

Filament bending flexibility is commonly-hypothesised as a source of asymmetry that might explain contraction of disordered actomyosin networks (Murrell and Gardel 2012; De La Cruz and Gardel 2015; du Roure et al. 2019; Head et al. 2003; Tam et al. 2021). Actin filaments are semi-flexible (Stachowiak et al. 2014; Belmonte et al. 2017), such that they undergo small but significant bending (Broedersz and Mackintosh 2014; Murrell and Gardel 2012). Filament semi-flexibility is irrelevant in sarcomeres with parallel arrays of straight filaments, but is important for disordered networks in which motors can cross-link filaments at arbitrary angles and generate torque. Previous experimental and theoretical studies in disordered networks suggest that filament buckling gives rise to contraction. These studies show that filaments can sustain longitudinal tension, but buckle under longitudinal compression (Bidone et al. 2017; Belmonte et al. 2017; Cheffings et al. 2016; du Roure et al. 2019; Freedman et al. 2017, 2018; Lenz 2020; Murrell and Gardel 2012; Ronceray et al. 2016; Soares e Silva et al. 2011; Yu et al. 2018). This buckling mechanism can generate network-scale bias to contraction over expansion (Belmonte et al. 2017). Other studies have considered a related filament bending mechanism (Lenz 2014; Tam et al. 2021; Head et al. 2003; Popov et al. 2016; Kim 2015; Letort et al. 2015) as a source of force asymmetry. Bending involves applying forces that pluck filaments transversely, in contrast to the longitudinal forces involved with buckling. Lenz (2014) showed that filament bending produces forces that exceed those involved with longitudinal buckling, and Tam et al. (2021) showed that this mechanism facilitates network-scale contraction. A pertinent question is whether the force asymmetry provided by bending or buckling applies at the microscopic scale (Lenz 2014; Komianos and Papoian 2018; De La Cruz and Gardel 2015), or whether long-range effects transmit contractile force through the network, without requiring a microscopic asymmetry (Ronceray et al. 2016).

One approach to understand microscopic filament dynamics is to model a single filament as an inextensible elastic rod, as in ‘worm-like chain’ models (Broedersz and Mackintosh 2014; Lenz 2014). Broedersz and Mackintosh (Broedersz and Mackintosh 2014) used this approach to identify an asymmetry under extension and compression. Other authors have considered structures consisting of two-filaments and an attached motor (Lenz 2014; Belmonte et al. 2017; Hiraiwa and Salbreux 2016; Komianos and Papoian 2018). Lenz (2014) reported that disordered networks of rigid filaments with polarity-reversal symmetry (*i.e.* any configuration of filaments is equally likely as the same configuration with minus and plus-ends reversed) generate zero net contraction. Lenz (2014) also showed that filament bending gives rise to contraction in a two-filament system, and is the dominant mechanism of contraction for experimentally-feasible parameters. During motor-induced deformation, semi-flexible actin filaments evolve to minimise their bending energy. Therefore, we model semi-flexible filament evolution as a curve-straightening flow. Mathematically, curve-straightening refers to deformation of curves in $\mathbb {R}^2$ by decreasing their total squared curvature. Curve-straightening problems have been investigated since the 1980s (Langer and Singer 1984, 1987; Linnér 1989, 2003). Wen (1993, 1995) used the indicatrix representation and $L^2$-gradient flow of the squared curvature functional to derive a fourth-order, semilinear parabolic partial differential equation (PDE) for the evolution of the curve. Oelz (2011) extended this work to model an open curve. However, theoretical analysis of curve-straightening flows is mostly limited to single curves, rather than the two-curve representations necessary to model a pair of filaments.

We extend previous curve-straightening models to derive a coupled PDE system for two symmetric, semi-flexible filaments with a myosin motor attached at their intersection. After obtaining the governing equations, we describe how to obtain the stress tensor, assuming the two filaments reside in a homogeneous background material. We then use asymptotic analysis and numerical solutions to provide a detailed explanation of how filament bending facilitates contraction on the two-filament scale. Our analysis suggests a contraction mechanism based neither on filament buckling, nor intrinsic force asymmetry where bending generates contraction. Instead, filament semi-flexibility creates a geometric asymmetry that inhibits expansion. Rigid filaments exhibit polarity-reversal symmetry, whereby contraction associated with a minus-end-located motor balances with expansion associated with a plus-end-located motor. Allowing filaments to bend breaks this symmetry, and the filaments become closer to parallel as the motor approaches the plus-ends. This decreases the spring force through the motor, enabling the motor to move faster close to the plus-ends. Fast motor motion inhibits expansive stress, and gives rise to net contraction. Our analysis provides a new hypothesis for bending-induced actomyosin contraction, and shows how contraction can occur on the microscopic two-filament scale.

## Mathematical model

**Fig. 1:**
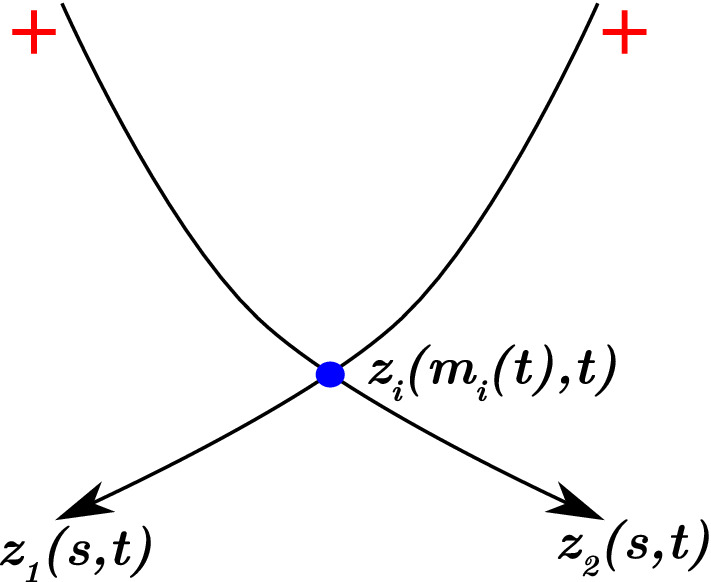
Schematic representation of two actin filaments with a myosin motor attached at their intersection. Filaments are the curves $z_1$ and $z_2,$ and arrow heads represent minus-ends. The myosin motor protein is represented by the blue dot

We develop a mathematical model for a myosin motor attached to two overlapping actin filaments. We represent filaments as open curves in $\mathbb {R}^2,$ and denote their positions by $z_i(s(t), t) = (x_i(s(t),t), y_i(s(t), t)),$ for $i = 1, 2$ (see Fig. 1). The variable $t$ denotes time, and $s \in [0, L_i]$ is the arc length parameter, where $L_i$ is the length of the $i$-th filament. Actin filaments are polarised, so we adopt the convention that $s = 0$ corresponds to the filament minus-end, and $s = L_i$ corresponds to the plus-end. Since non-muscle myosin thick filaments are short compared to actin filaments (Dasbiswas et al. 2018), we represent the myosin motor as a point object existing at the intersection between the two filaments. We track its position by introducing the variables $m_i(t) \in [0, L_i],$ such that $s = m_i$ is the position of the motor head attached to the $i$-th filament. We assume that no other proteins, for example cross-linkers, are present.

### Discrete force-balance equations

We express the mathematical model for the filament and motor mechanics as a system of force-balance equations,

2.1 $$\begin{aligned} F_{a, \mathrm {drag}} - \delta E_{a,\mathrm {bend}} - \delta E_{a,\mathrm {stretch}} - \delta E_{m,\mathrm {stretch}} + F_{m,a} = {\varvec{{0}}}. \end{aligned}$$

The first three terms in (2.1) describe the drag, bending, and longitudinal stretching forces respectively on actin filaments. The fourth term represents longitudinal stretching along the myosin motor, and the final term describes forces between filaments and motors. We represent bending and stretching forces as the variation of potential energy, where terms involving $\delta$ denote variations. We formulate the force-balance equations (2.1) as a minimisation problem. This involves constructing a time-discrete scalar functional that contains a contribution for each force term in (2.1),

2.2 $$\begin{aligned} E\left[ z_1, z_2, m_1, m_2\right] := E_{a, \mathrm {drag}} + E_{a,\mathrm {bend}} + E_{a,\mathrm {stretch}} + E_{m,\mathrm {stretch}} + E_{m,a}. \end{aligned}$$

In (2.2), $E_{a,\mathrm {bend}},$ $E_{a,\mathrm {stretch}},$ and $E_{m,\mathrm {stretch}}$ are the potential energies associated with filament bending, filament stretching, and motor stretching respectively. The terms $E_{a, \mathrm {drag}}$ and $E_{m,a}$ are pseudo-energy terms with variations that correspond to finite-difference approximations of $F_{a, \mathrm {drag}}$ and $F_{m,a},$ which cannot be interpreted as variations of potential energy.

The first term, $E_{a, \mathrm {drag}},$ describes drag friction between filaments and a passive background medium. Drag acts uniformly along the filaments and opposes filament motion. The term to represent drag between filaments and the background medium is

2.3 $$\begin{aligned} E_{a, \mathrm {drag}} = \lambda _a\sum _{i=1}^{2} \int _0^{L_i} \frac{\left|z_i - z_i^{n}\right|^2}{2\Delta t} \,\text {d}s, \end{aligned}$$

where $\lambda _a$ is the filament drag coefficient, $\Delta t$ is the time step size, and the superscript $n$ refers to the previous time step in the discrete formulation, $z_i^n = z_i(s, t-\Delta t).$ We model bending of semi-flexible actin filaments via the elastic potential energy

2.4 $$\begin{aligned} E_{a, \mathrm {bend}} = \sum _{i=1}^{2} \int _0^{L_i} \frac{\kappa _a}{2}\left|z_i'' \right|^2 \,\text {d}s, \end{aligned}$$

where $\kappa _a$ is the flexural rigidity, and primes denote differentiation with respect to arc-length, $s.$ We assume that $\kappa _a$ is constant, and the same for both filaments. We obtain a term for filament stretching by assuming that actin filaments are inextensible. To model this, we ensure that $|z_i'|= 1$ at every point along the filaments using the penalisation term

2.5 $$\begin{aligned} E_{a, \mathrm {stretch}} = \sum _{i=1}^{2} \int _0^{L_i}\frac{1}{\delta _a}\left( \left|z_i'\right|- 1\right) ^2\,\text {d}s, \end{aligned}$$

where $\delta _a$ is an arbitrarily small parameter that enforces the inextensibility constraints.

**Fig. 2:**
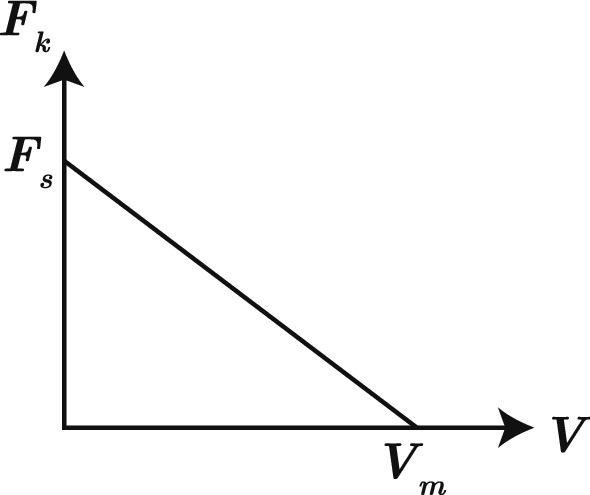
The linear force–velocity relationship for myosin motors bound to actin filaments. An unextended motor subject to zero force moves towards filament plus-ends with the free-moving velocity, $V_m.$ If the spring force through the motor exceeds the stall force $F_s,$ the motor does not move

The remaining two terms in (2.2) describe how motors contribute to the mechanics. To model motor stretching, we introduce another penalising potential,

2.6 $$\begin{aligned} E_{m, \mathrm {stretch}} = \frac{1}{\delta _m} \left|z_1(m_1, t) - z_2(m_2, t) \right|^2, \end{aligned}$$

where $\delta _m$ is an arbitrarily small parameter that penalises deviation from the constraint $z_1(m_1, t) = z_2(m_2, t)$ stating that motors are to remain point objects. The final term in (2.2) describes interactions between filaments and motors. We assume that motors obey a linear (affine) force–velocity relation (Alcazar et al. 2019), illustrated in Fig. 2. This law integrates the multitude of force contributions exerted by myosin heads which decorate the myosin thick filament and interact with actin filaments (Kull and Endow 2013). Subject to zero force (when the motor is unextended), motors move with speed $V_m.$ As the motor extends, the spring force through the motor increases. We assume that motor velocity varies linearly with the spring force through the motor. If the force through the motor exceeds $F_s,$ the stall force, then the motor velocity is zero. The corresponding pseudo-energy term consists of a linear term, and a quadratic drag-like term for the velocity reduction caused by the force through the motor,

2.7 $$\begin{aligned} E_{m, a} = \sum _{j=1}^{2} \left( -F_s \, m_{j} + \frac{F_s}{V_m}\frac{\left( m_{j} - m_{j}^n\right) ^2}{2\Delta t} \right) . \end{aligned}$$

With the definition of (2.3-2.7), minimising the functional (2.2) for fixed $\Delta t$ provides a time-implicit numerical method to solve the force-balance equations (2.1) for the filament and motor positions.

### Governing partial differential equations

Formulating the model as a minimisation problem enables us to derive a continuum model for the filament and motor positions based on the discrete formulation in Sect. 2.1. The derivation is based on the following variational principle. Given known data $(z_1^{n}, z_2^{n}, m_1^{n}, m_2^{n})$ at the discrete point in time $n,$ the solution at the following point in time minimises the functional (2.2), that is

2.8 $$\begin{aligned} \left( z_1^{n+1}, z_2^{n+1}, m_1^{n+1}, m_2^{n+1}\right) = {{\,\mathrm{\arg \!\min }\,}}E\left[ z_1^{n}, z_2^{n}, m_1^{n}, m_2^{n}\right] \left( z_1, z_2, m_1, m_2\right) . \end{aligned}$$

We obtain force-balance equations by setting to zero the functional derivatives of (2.2) with respect to filament and motor positions. Subsequently, we write

2.9 $$\begin{aligned} \delta E\left[ z_1^{n}, z_2^{n}, m_1^{n}, m_2^{n}\right] \left( z_1^{n+1}, z_2^{n+1}, m_1^{n+1}, m_2^{n+1}\right) \cdot \left( \delta z_1, \delta z_2, \delta m_1, \delta m_2\right) = 0, \end{aligned}$$

where terms involving $\delta$ denote the variation of the respective quantity. Minimising the functional (2.2) enables us to write the force-balance equations (2.1) in terms of $z_1,$ $z_2,$ $m_1,$ and $m_2.$ We obtain the governing equations by evaluating (2.9) and matching coefficients of $\delta z_1,$ $\delta z_2,$ $\delta m_1,$ and $\delta m_2.$ On taking the formal continuum limit $\Delta t \rightarrow 0,$ for which $(u - u^n)/\Delta t \rightarrow \dot{u},$ we obtain the system of PDEs

2.10a $$\begin{aligned}&\lambda _{a}\dot{z_1} + \kappa _{a}z_1'''' - \left( \lambda _1z_1'\right) ' + \mu \frac{z_1-z_2}{\Vert z_1-z_2\Vert } \delta (s-m_1) = 0, \end{aligned}$$

2.10b $$\begin{aligned}&\lambda _{a}\dot{z_2} + \kappa _{a}z_2'''' - \left( \lambda _2z_2'\right) ' - \mu \frac{z_1-z_2}{\Vert z_1-z_2\Vert } \delta (s-m_2) = 0, \end{aligned}$$

2.10c $$\begin{aligned}&\dot{m_1} = V_m\left[ 1 - \frac{\mu }{F_s}\frac{z_1-z_2}{\Vert z_1-z_2\Vert }\cdot z_1'(m_1,t)\right] , \end{aligned}$$

2.10d $$\begin{aligned}&\dot{m_2} = V_m\left[ 1 + \frac{\mu }{F_s}\frac{z_1-z_2}{\Vert z_1-z_2\Vert }\cdot z_2'(m_2,t)\right] , \end{aligned}$$

where primes denote differentiation with respect to arc length, dots represent derivatives with respect to time, and $\delta (\cdot )$ is the Dirac delta function (not to be confused with variation). Equations (2.10) are a system of continuum force-balance equations for the filament and motor positions. They are formulated in a formal limit where $\delta _a$ and $\delta _m$ are small, and the force coefficients $1/\delta _a$ and $1/\delta _m$ in the variations of the penalising potentials (2.5) and (2.6) are replaced by the Lagrange multipliers $\lambda _1,$ $\lambda _2,$ and $\mu .$ Note that the sign of $z_1-z_2$ in (2.10) will be absorbed by $\mu .$ As a consequence, solutions satisfy the constraints

2.11a $$\begin{aligned}&|z_{i}'|\equiv 1, \end{aligned}$$

2.11b $$\begin{aligned}&z_1(m_1,t)=z_2(m_2, t). \end{aligned}$$

The equations are subject to the boundary and initial conditions 2.12a2.12b2.12c where the subscript $I$ represents an initial quantity. A detailed derivation of (2.10) and (2.12) is provided in Appendix A.

### Calculation of forces and stress

**Fig. 3:**
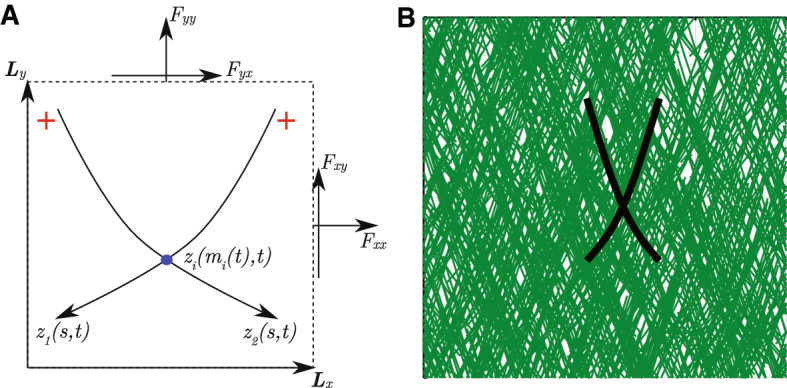
Schematic representation of a two-filament–motor system existing within a rectangular homogeneous background medium. (A) The filaments are the curves $z_1$ and $z_2,$ and arrow heads represent filament minus (pointed) ends. Myosin motor proteins are represented by blue dots, and initially appear at the intersection between the two filaments. (B) Visualisation of the two-filament–motor structure embedded in a dense background network consisting of numerous assemblies (green lines) with the same shape as the reference pair (black lines)

An objective of this work is to describe how the filament and motor motion governed by our model generates contractile and expansive forces. We assume the two-filament–motor structure is immersed in a dense network of cross-linked filaments covering a rectangular domain. A pair of actin filaments can locally deform the background network in which it is immersed. However, we assume the background can only undergo uniform elongation and shearing. A scenario supporting this assumption is that the background network consists of numerous two-filament–motor assemblies, all with the same shape as the reference pair that we describe explicitly (Fig. 3B). In this scenario, deformations occur equally everywhere in the domain, and the background network remains homogeneous. If the background is homogeneous, we can associate the tension at the boundaries of the domain with the stress being generated by the reference pair of actin filaments (Fig. 3A).

To quantify contraction, we compute the stress tensor for a small rectangular region of background material that encloses the two-filament–motor structure. The adjacent sides of the rectangle are given by the vectors ${\varvec{{L}}}_{x} = (L_{xx}, L_{xy})^T,$ and ${\varvec{{L}}}_y = (L_{yx}, L_{yy})$ as shown in Fig. 3A. We compute the vectors ${\varvec{{F}}}_{x} = (F_{xx}, F_{xy})$ and ${\varvec{{F}}}_y = (F_{yx}, F_{yy}),$ also shown in Fig. 3A. These vectors are the force components acting on the domain boundaries that must be applied to prevent uniform elongation and shear deformations.

These forces sum the contributions of both filaments and the motor, and provide a measure of net contractility. To derive closed form expressions for these forces, we return to the discrete formulation. We obtain ${\varvec{{F}}}_x$ and ${\varvec{{F}}}_y$ by first adding extra terms to the functional (2.2), and defining

2.13 $$\begin{aligned} \begin{aligned} E_{\mathrm {total}}&:= \tilde{E}_{a, \mathrm {drag}} + E_{a,\mathrm {bend}} + E_{a,\mathrm {stretch}} + E_{m,\mathrm {stretch}} + E_{m,a} \\&+ {\varvec{{F}}}_x\cdot {\varvec{{L}}}_x + {\varvec{{F}}}_y\cdot {\varvec{{L}}}_y. \end{aligned} \end{aligned}$$

In (2.13), we use the modified drag term

2.14 $$\begin{aligned} \tilde{E}_{a, \mathrm {drag}} = \lambda _a\sum _{i=1}^{2} \int _0^{L_i} \frac{\left|z_i - Lz_i^{n}\right|^2}{2\Delta t} \,\text {d}s, \end{aligned}$$

where

2.15 $$\begin{aligned} L = \begin{bmatrix} L_{xx}/L_{xx}^n &{} L_{yx}/L_{yy}^n \\ L_{xy}/L_{xx}^n &{} L_{yy}/L_{yy}^n \end{bmatrix}. \end{aligned}$$

The matrix $L$ represents the transition from the coordinate frame ${\varvec{{L}}}^n_x,$ ${\varvec{{L}}}^n_y$ at time $n,$ to the new coordinate frame ${\varvec{{L}}}_x,$ ${\varvec{{L}}}_y,$ corresponding to a rectangle that has undergone uniform shearing and elongation. If we impose ${\varvec{{L}}}_x = {\varvec{{L}}}_x^n$ and ${\varvec{{L}}}_y = {\varvec{{L}}}_y^n$, the vectors  and  represent Lagrange multipliers that enforce the constant domain size and shape constraints. We assume that any possible deformations of the rectangle are small, such that Cauchy stress theory applies. The two-dimensional state of stress in the domain is then given by the stress tensor,

2.16 $$\begin{aligned} \varvec{{\sigma }} = \begin{bmatrix} F_{xx}/L_{yy} &{} F_{xy}/L_{yy} \\ F_{yx}/L_{xx} &{} F_{yy}/L_{xx} \end{bmatrix}. \end{aligned}$$

The bulk stress,

2.17 $$\begin{aligned} \sigma = \frac{1}{2}\mathrm {tr}\left( \varvec{{\sigma }}\right) , \end{aligned}$$

then provides a measure of the contraction or expansion generated by the two-filament–motor system. By convention, negative $\sigma$ indicates contraction, and positive $\sigma$ indicates expansion. The quantity $\sigma$ is invariant to domain rotations, and equal to the average of the eigenvalues of $\varvec{{\sigma }}.$ The associated eigenvectors of $\varvec{{\sigma }}$ are the principal stress directions, which indicate the directions of maximum contraction or expansion.

To obtain an explicit expression for the bulk stress, $\sigma ,$ in terms of the filament positions, we differentiate the functional (2.13) with respect to ${\varvec{{L}}}_x$ and ${\varvec{{L}}}_y.$ This yields

2.18a $$\begin{aligned} {\varvec{{F}}}_x&= \lambda _a\sum _{i=1}^{2}\int _0^{L_i} \frac{x_{i}^{n}}{L_{xx}^{n}}\frac{\left( z_i - Lz_{i}^{n}\right) }{\Delta t}\,\text {d}s, \end{aligned}$$

2.18b $$\begin{aligned} {\varvec{{F}}}_y&= \lambda _a\sum _{i=1}^{2}\int _0^{L_i} \frac{y_{i}^{n}}{L_{yy}^{n}}\frac{\left( z_i - Lz_{i}^{n}\right) }{\Delta t}\,\text {d}s. \end{aligned}$$

Applying the formal continuum limit $\Delta t \rightarrow 0,$ $L\rightarrow \varvec{{I}},$ and $(z_i - z_i^n)/\Delta t \rightarrow \dot{z}_i,$ we obtain 2.19a2.19b Evaluating the bulk stress (2.17) then yields2.20Furthermore, the expressions (2.19) confirm that2.21where $z_i^\perp$ denotes a vector orthogonal to $z_i,$ and we obtain the result by substituting (2.10) for $\dot{z}_i.$ The stress tensor (2.16) is thus symmetric, and the bulk stress $\sigma$ is equal to the average of the eigenvalues of $\varvec{{\sigma }}.$

### Nondimensionalisation

We nondimensionalise the PDE model (2.10)-(2.12) by introducing the length and time scales

2.22 $$\begin{aligned} \hat{t} = \frac{F_s}{\lambda _a L_a^2}t, \quad \text { and }\quad \left( \hat{x}, \hat{y}\right) = \frac{1}{L_a}\left( x, y\right) , \end{aligned}$$

where hats represent dimensionless variables, and $L_a$ is a characteristic filament length. The dimensionless model is then (dropping hats for convenience)

2.23a $$\begin{aligned}&\dot{z_1} + \kappa ^*z_1'''' - \left( \lambda ^*_1z_1'\right) ' + \mu ^*\frac{z_1-z_2}{\Vert z_1-z_2\Vert }\delta ^*(s-m_1) = 0, \end{aligned}$$

2.23b $$\begin{aligned}&\dot{z_2} + \kappa ^*z_2'''' - \left( \lambda ^*_2z_2'\right) ' - \mu ^*\frac{z_1-z_2}{\Vert z_1-z_2\Vert }\delta ^*(s-m_2) = 0, \end{aligned}$$

2.23c $$\begin{aligned}&\frac{1}{V_m^*}\dot{m_1} = 1 - \mu ^*\frac{z_1-z_2}{\Vert z_1-z_2\Vert }\cdot z_1'(m_1,t), \end{aligned}$$

2.23d $$\begin{aligned}&\frac{1}{V_m^*}\dot{m_2} = 1 + \mu ^*\frac{z_1-z_2}{\Vert z_1-z_2\Vert }\cdot z_2'(m_2,t), \end{aligned}$$

subject to the boundary and initial conditions 2.24a2.24b2.24c and the constraints

2.25a $$\begin{aligned}&|z_{i}'|\equiv 1, \end{aligned}$$

2.25b $$\begin{aligned}&z_1(m_1,t)=z_2(m_2, t), \end{aligned}$$

where $\delta ^*(\hat{x}) = L_a\delta (L_a \hat{x})$ is a scaled Dirac delta function. The dimensionless parameters and forces are

2.26 $$\begin{aligned} \begin{aligned} \kappa ^* = \frac{\kappa }{F_sL_a^2}, \quad \lambda _i^* = \frac{\lambda _i}{F_s}, \quad \mu _i^* = \frac{\mu _i}{F_s}, \\ V_m^* = \frac{V_m\lambda _aL_a}{F_s}, \quad \text { and }\quad L_i^* = \frac{L_i}{L_a}. \end{aligned} \end{aligned}$$

### Model simplification

Before obtaining asymptotic and numerical results, we consider a simplification to the dimensionless model (2.23)-(2.25). First, we assume that the two filaments are symmetric about the vertical, that is

2.27 $$\begin{aligned} z_1 = z, \quad z_2 = \begin{bmatrix} -1 &{} 0 \\ 0 &{} 1\end{bmatrix}z, \end{aligned}$$

and have identical length $L_a = L_1 = L_2.$ Symmetry also implies that the relative position of the motor is the same for both filaments, $m_1 = m_2 = m,$ and that $\lambda ^*_1 = \lambda ^*_2 = \lambda ^*.$ To simplify the motor dynamics (2.23c)-(2.23d), we impose $V_m \rightarrow \infty .$ Finally, we rewrite dimensionless flexural rigidity according to $\kappa ^* = 1/\varepsilon ,$ indicating that the flexural rigidity is large ($\varepsilon \ll 1$), and that the filaments undergo only minor bending. On applying these simplifications, the dimensionless model (2.23) becomes (dropping asterisks on dimensionless parameters) 2.28a2.28b subject to the boundary and initial conditions 2.29a2.29b2.29c and the constraints

2.30a $$\begin{aligned}&|z'|= 1, \end{aligned}$$

2.30b $$\begin{aligned}&z(m(t),t) =\begin{pmatrix} 0 \\ y(t) \end{pmatrix}. \end{aligned}$$

Equations (2.28a) and (2.28b) describe the filament and motor evolution respectively, under the simplifying assumptions. Equations (2.28a) and (2.28b), with the boundary and initial conditions (2.29), and constraints (2.30), complete our simplified model.

The dimensionless bulk stress (2.20) for the simplified model becomes2.31To obtain a measure of net stress, we integrate $\sigma$ over the time between motor attachment and detachment. This yields

2.32 $$\begin{aligned} \int _{0}^{T} \sigma \,\text {d}t = J(T) - J(0), \end{aligned}$$

where

2.33 $$\begin{aligned} J(t) = \int _{0}^{1} \left|z(s,t) \right|^2 \,\text {d}s. \end{aligned}$$

The quantity $J(T) - J(0)$ describes the net, time-aggregated stress that the two filaments produce between motor attachment and detachment. This quantity will be important in our asymptotic and numerical investigation on how filament bending affects contraction.

## Results and discussion

We analyse the simplified model derived in Sect. 2.5 to quantify how filament flexibility gives rise to contractile stress. First, we use asymptotic analysis to obtain an explicit approximation to the solution in the limit of infinite flexural rigidity. Through the leading-order problem, we show that a rigid two-filament–motor structure with polarity-reversal symmetry produces zero net stress. The first-order problem gives rise to a system of differential equations that governs the dynamics with small filament bending. Second, we obtain numerical solutions to validate the asymptotic analysis, and investigate solutions beyond the large flexural rigidity limit. These solutions reveal that contraction arises from a geometric asymmetry, whereby filaments become more parallel as the motor approaches the plus-ends. This inhibits expansion associated with plus-end-located myosin motors. Since contraction associated with minus-end-located motors is unaffected, the net outcome is a contractile two-filament–motor structure.

### Asymptotic analysis

We construct an asymptotic approximation to the solution of the simplified symmetric model (2.28)-(2.30). Asymptotic analysis involves expanding variables in powers of $\varepsilon ,$

3.1a $$\begin{aligned} z&= z_0 + \varepsilon z_1 + \varepsilon ^2 z_2 + \mathcal {O}(\varepsilon ^3), \end{aligned}$$

3.1b $$\begin{aligned} m&= m_0 + \varepsilon m_1 + \varepsilon ^2 m_2 + \mathcal {O}(\varepsilon ^3),\end{aligned}$$

3.1c $$\begin{aligned} \lambda&= \lambda _0 + \varepsilon \lambda _1 + \varepsilon ^2\lambda _2 + \mathcal {O}(\varepsilon ^3),\end{aligned}$$

3.1d $$\begin{aligned} \mu&= \mu _0 + \varepsilon \mu _1 + \varepsilon ^2\mu _2 + \mathcal {O}(\varepsilon ^3),\end{aligned}$$

3.1e $$\begin{aligned} \sigma&= \sigma _0 + \varepsilon \sigma _1 + \varepsilon ^2\sigma _2 + \mathcal {O}(\varepsilon ^3),\end{aligned}$$

3.1f $$\begin{aligned} J&= J_0 + \varepsilon J_1 + \varepsilon ^2J_2 + \mathcal {O}(\varepsilon ^3), \end{aligned}$$

as $\varepsilon \rightarrow 0.$ On substituting the asymptotic series (3.1) into the model (2.28)-(2.30), the leading-order solution is the evolution of two rigid filaments with infinite resistance to bending. The first-order problem describes how small, non-zero bending affects the dynamics and stress. We present the key results and arguments in subsequent subsections, and give full details of the computations in Appendix B.

#### Leading-order solution

**Fig. 4:**
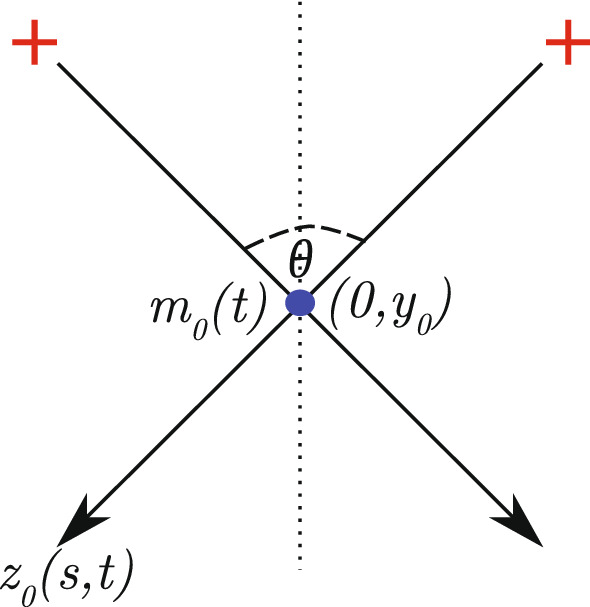
Schematic of a two-filament–motor system with rigid symmetric actin filaments. The myosin motor has relative position $m_0(t),$ and physical position $(0, y_0(t)).$ Filaments are symmetric about the dashed vertical line, which is the positive $y$-axis. The angle between the filaments is $\theta ,$ such that the angle between a filament and the $y$-axis is $\theta /2.$

The leading-order solution describes the evolution of rigid filaments. To solve for $z_0,$ we consider the balance at $\mathcal {O}(1/\varepsilon ).$ This yields3.2The solution to the $\mathcal {O}(1/\varepsilon )$ problem (3.2) is a straight filament, whose direction we parameterise by the filament angle, $\theta /2,$ measured from the positive vertical axis (see Fig. 4). We write

3.3 $$\begin{aligned} z_0'= \begin{bmatrix} \sin \left( \theta /2\right) \\ \cos \left( \theta /2\right) \end{bmatrix}. \end{aligned}$$

For a filament orthogonal to $z_0$ we use the notation

3.4 $$\begin{aligned} z_0'^\perp = \begin{bmatrix} -\cos \left( \theta /2\right) \\ \sin \left( \theta /2\right) \end{bmatrix}, \end{aligned}$$

where the symbol $^\perp$ denotes rotation to the left by $\pi /2$. A suitable ansatz for the position of a rigid filament solution satisfying the constraint (2.30b) is then

3.5 $$\begin{aligned} z_0 = \begin{pmatrix} 0 \\ y_0 \end{pmatrix} + z_0'\left( s - m_0\right) , \end{aligned}$$

where the leading-order motor relative position, $m_0,$ and leading-order vertical position of the intersection, $y_0,$ complete the parameterisation. The leading-order ansatz is illustrated in Fig. 4.

To obtain the leading-order solution, we consider the $\mathcal {O}(1)$ problem 3.6a3.6b3.6c3.6d3.6e Equation (3.6a)-(3.6b) are the leading-order equations governing the filaments and motor respectively. Equation (3.6c) provides two boundary conditions, and the solution must also satisfy the ansatz (3.6d) and orthogonality constraint (3.6e). To proceed, we use the orthogonality condition (3.6e) to infer the ansatz

3.7 $$\begin{aligned} z_1'(s,t) = h'(s,t)z_0'^\perp , \end{aligned}$$

where $h(s,t)$ is an arbitrary scalar function. Substituting the ansatzes (3.5) and (3.7) into the PDE for filament evolution (3.6a) enables us to solve for the leading-order quantities

3.8 $$\begin{aligned} \mu _0 = \frac{1}{\sin (\theta /2)}, \quad \lambda _0 = H\left( s-m_0\right) - s, \quad \text { and }\quad \sigma _0 = 2\nu _0, \end{aligned}$$

where $H$ is the Heaviside step function, and $\nu _0 = m_0 - 1/2.$ Filament evolution then satisfies the ordinary differential equations

3.9a $$\begin{aligned} \frac{\text {d}S}{\text {d}t}&= -24\nu _0\left( 1-S\right) , \end{aligned}$$

3.9b $$\begin{aligned} S\frac{\text {d}\nu _0}{\text {d}t}&= 1 + 12\nu _0^2\left( 1-S\right) , \end{aligned}$$

where $S = \sin ^2(\theta /2).$ Since $z_0$ is written in terms of the angle $\theta$ only, the system (3.9) determines $z_0.$ With $h'$ known, we subsequently obtain $z_1.$ Full details on this calculation are available in Appendix B.1.

An important property of the system (3.9) is that it is invariant under a change of variables that reverses the direction of time. If we introduce the reversed-time $\tilde{t} = T-t$ for an arbitrary constant $T,$ we have $\tilde{\nu }_0(\tilde{t}) = -\nu _0(T - \tilde{t})$ and $\tilde{\theta }(\tilde{t})=\theta (T - \tilde{t}),$ and subsequently $\tilde{S} = S.$ Consequently, if the motor is initially positioned at the pointed ends ($\nu _0(0)=-1/2$), and $T$ denotes the time it reaches the barbed ends, then the time-aggregated stress vanishes,

3.10 $$\begin{aligned} J(T)-J(0)&= \int _0^{T/2} \sigma (t) \,\text {d}t +\int _{T/2}^T \sigma (t) \,\text {d}t,\nonumber \\&=\int _0^{T/2} 2 \nu _0(t) \,\text {d}t + \int ^{T/2}_0 2 \nu _0(T-\tilde{t}) \,\text {d}\tilde{t}\nonumber \\&=\int _0^{T/2} 2 \nu _0 \,\text {d}t - \int ^{T/2}_0 2 \tilde{\nu }_0 \,\text {d}\tilde{t} = 0. \end{aligned}$$

**Fig. 5:**
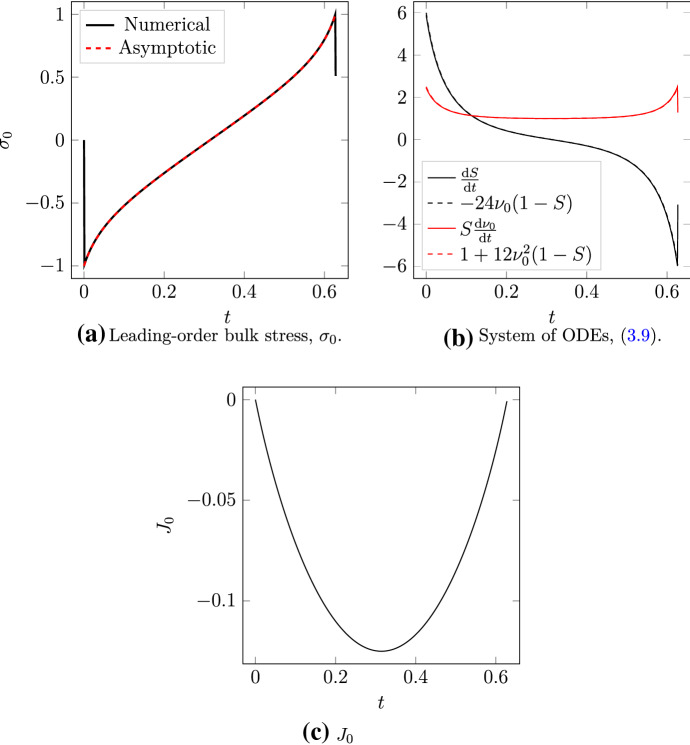
Numerical results for a solution with two rigid filaments ($\varepsilon = 1 \times 10^{-5}$). **A** Comparison between the numerical bulk stress, and the leading-order approximation given by (3.8). **B** Numerical validation of the system of ODEs (3.9). **C** Numerical result for $J(t),$ confirming that $J(T) - J(0) = 0$ for rigid filaments

This is because the equations and initial conditions satisfied by $\nu _0,$ $\theta ,$ and $\tilde{\nu }_0,$ $\tilde{\theta }$ both coincide, and we have that $\nu _0(\hat{t})=\tilde{\nu }_0(\hat{t})$ for all $\hat{t} \in [0,T]$ (see also numerical result shown in Fig. 5C). This finding agrees with the previously reported results that rigid filaments with polarity-reversal symmetry produce zero net stress (Dasanayake et al. 2011; Lenz 2014).

#### First-order correction

The higher-order correction terms, $z_1,$ $\sigma _1$ and $J_1,$ elucidate the effect of small, non-zero bending on filament evolution and stress. To obtain expressions for the first-order corrections to bulk stress and $J,$ we substitute the asymptotic expansions (3.1) into the stress terms (2.31) and (2.32). This yields

3.11a $$\begin{aligned} \sigma&= -2\int _{0}^{1} \lambda _0 \,\text {d}s - 2\varepsilon \int _{0}^{1} \left( \left|z_1''\right|^2 + \lambda _1 \right) \,\text {d}s + \mathcal {O}\left( \varepsilon ^2\right) , \end{aligned}$$

3.11b $$\begin{aligned} J&= \int _{0}^{1} \left|z_0\right|^2 \,\text {d}s + 2\varepsilon \int _{0}^{1} z_0\cdot z_1 \,\text {d}s + \mathcal {O}\left( \varepsilon ^2\right) . \end{aligned}$$

Matching coefficients of $\varepsilon$ then yields

3.12 $$\begin{aligned} \sigma _1 = -2\int _{0}^{1} \left( \left|z_1''\right|^2 + \lambda _1 \right) \,\text {d}s, \quad J_1 = 2\int _{0}^{1} z_0\cdot z_1 \,\text {d}s. \end{aligned}$$

In addition, we use the PDE (3.6a) and the ansatz (3.7) to obtain an explicit expression for the curvature of $z_1,$

3.13 $$\begin{aligned} h'' = -\cot \left( \frac{\theta }{2}\right) \left[ (m_0 - s) H(s - m_0) + s^2(m_0(2s - 3) - s + 2)\right] . \end{aligned}$$

Since the first-order correction to stress, $\sigma _1,$ involves the currently unknown $\lambda _1,$ progress requires consideration of the $\mathcal {O}(\varepsilon )$ problem, which is 3.14a3.14b3.14c3.14d3.14e3.14f Obtaining the solution to (3.14) involves an intricate calculation based on the ansatz

3.15 $$\begin{aligned} z_1= \begin{pmatrix} 0 \\ y_1(t) \end{pmatrix} - z_0'(t)m_1 + z_0'^\perp \left[ A(t)(s-m_0) + \int _{m_0}^s \tilde{h}'(s,t)\,\text {d}s \right] , \end{aligned}$$

where $A(t)$ is a (possibly time-dependent) constant of integration. The form of (3.15) arises from the ansatzes (3.14e) and (3.7), and gives rise to a system of equations for the degrees of freedom $A(t),$ $y_1(t),$ and $m_1(t).$ We provide full details on the calculation to obtain this in Appendix B.2. A key result is the stress correction term,

3.16 $$\begin{aligned} \sigma _1 = -2\int _{0}^{1} \left|h''\right|^2 \,\text {d}s -2 \left[ A + \tilde{h}'(m_0,t) \right] \cot \left( \frac{\theta }{2} \right) \left( \frac{1}{2}-m_0 \right) + 2m_1, \end{aligned}$$

where $\tilde{h}'(m_0,t)$ is given by

3.17 $$\begin{aligned} \tilde{h}'(m_0,t)=-\frac{1}{12} m_0^3 \left( 6 m_0^2-15 m_0+8\right) \cot \left( \frac{\theta }{2}\right) . \end{aligned}$$

Similar to the system (3.9), we can obtain a system of differential equations to solve for $A(t),$ $y_1(t),$ and $m_1(t).$ Since $h''$ and $\tilde{h}'$ are in terms of the leading-order degrees of freedom $\theta$ and $m_0,$ we can subsequently compute $\sigma _1.$ However, the ODEs for $A(t),$ $y_1(t),$ and $m_1(t)$ have no exact solution. Therefore, we continue our investigation using numerical solutions.

### Numerical solutions

We compute numerical solutions to the simplified governing equations for filament and motor positions (2.28) in Julia. Our numerical method involves minimising the time-discrete functional (2.13) with $\Delta t = 0.001.$ Energy minimisation is equivalent to a time-implicit numerical method for solving the dimensionless model (2.23). In our solutions, each filament has total length 1${\upmu }\hbox {m}$ (Kamasaki et al. 2007), and consists of 50 equal-length line segments joined at nodes, about which segments can rotate. In Julia, we use the package Optim.jl (Mogensen and Risbeth 2018) to obtain the minimiser using the limited-memory Broyden–Fletcher–Goldfarb–Shanno (LBFGS) method. After obtaining the minimiser, at each time step we use automatic differentiation (ForwardDiff.jl) of the functional (2.13) to compute the forces ${\varvec{{F}}}_x$ and ${\varvec{{F}}}_y,$ and subsequently bulk stress $\sigma$ (2.17).

#### Comparison with asymptotic analysis

We begin by computing numerical solutions for two symmetric filaments with $m(0) = 0,$ and $\theta (0) = \pi /2.$ Like the asymptotic analysis, we assume these filaments are initially rigid, $V_m \rightarrow \infty ,$ and solve until the motor reaches the plus-end and detaches. First, we compute a solution for two rigid ($\varepsilon = 1\times 10^{-5}$) filaments, to validate the leading-order bulk stress $\sigma _0 = 2\nu _0,$ and the solution to the system of ODEs (3.9), which governs $z_0.$ As Fig. 5A and B show, for both of these we obtain agreement between the numerical solution and leading-order solution. Furthermore, Fig. 5C illustrates the result from (3.10), namely that zero net stress is generated when a motor traverses two rigid filaments from the minus to plus-ends, *i.e.* $J_0(T) = J_0(0) = 0,$ where $T = 0.627$ is the time at which the motor reaches the plus end.

**Fig. 6:**
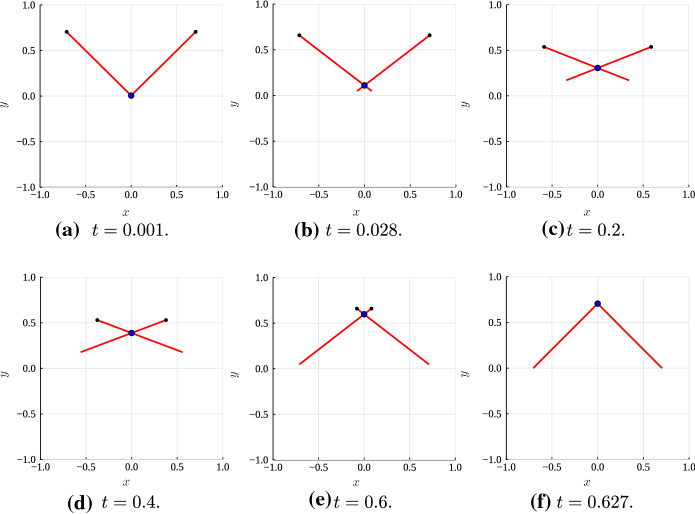
Numerical solution for the evolution of two actin filaments (red solid curves) with $\varepsilon = 0.01.$ The black nodes indicate the filament plus ends, and the blue dot at the filament intersection represents the myosin motor. **A** $t = 0.001$; **B** $t = 0.028.$; **C** $t = 0.2.$; **D** $t = 0.4.$ (E) $t = 0.6.$ (F) $t = 0.627.$

Next, we solve the model with $\varepsilon = 0.01$ to validate the formulae for $h''$ and $\sigma _1,$ (3.13) and (3.16) respectively. The dynamics of the two filaments and motor are illustrated in Fig. 6. As part of the solution, we compute $h''$ using the asymptotic formula (3.13) and numerical values of $\theta$ and $m,$ and compare this with the numerical value for the curvature,

3.18 $$\begin{aligned} h'' = \frac{1}{\varepsilon }\left( z_0'^\perp \cdot z''\right) . \end{aligned}$$

**Fig. 7:**
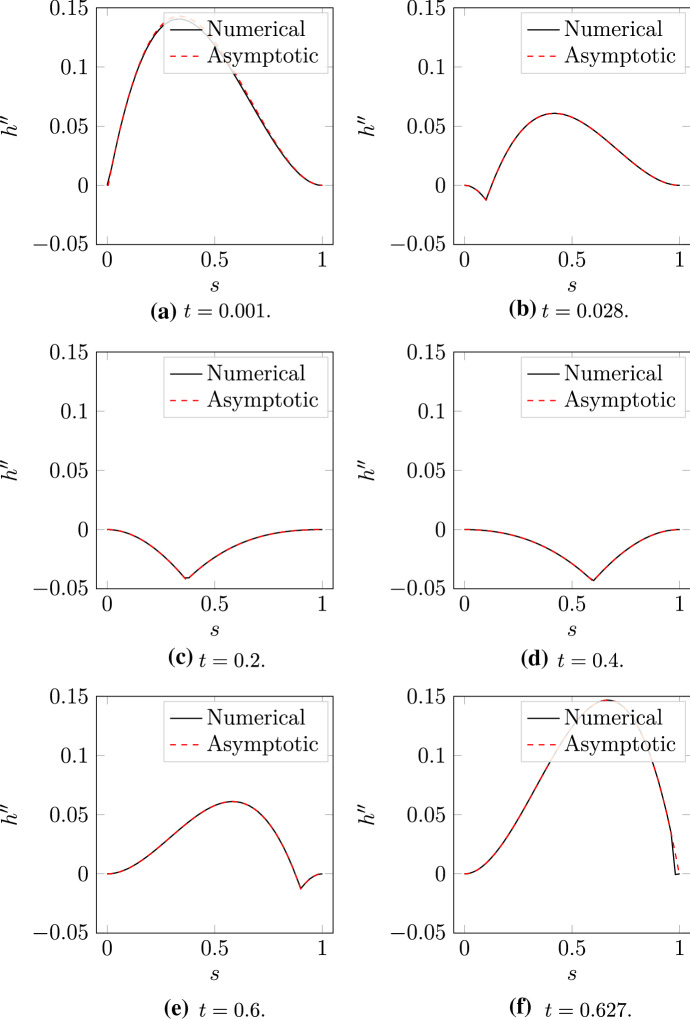
Numerical (black solid curves) and asymptotic (red dashed curves) solutions for $h''(s,t),$ the curvature of $z_1,$ in a numerical solution with $\varepsilon = 0.01,$ and $\theta (0) = \pi /2.$ **A** $t = 0.001$; **B** $t = 0.028.$; **C** $t = 0.2.$; **D** $t = 0.4.$; **E** $t = 0.6.$; **F** $t = 0.627.$

As Fig. 7 shows, we obtain agreement between the numerical and asymptotic results. The curvature formula (3.13) also reveals the shape that the two filaments adopt as they evolve (the qualitative pattern is easier to see in Fig. 10). Initially, the filaments adopt a convex shape, as the positive curvature in Fig. 7A shows. As the motor moves and pulls the filaments inwards, their shape changes to concave, as Figs. 7C–D show. When the motor approaches the plus-end, the filaments return to a convex shape. The asymptotic result for $h''$ remains accurate for up to $\varepsilon \thicksim \mathcal {O}(1),$ before breaking down for $\varepsilon \thicksim \mathcal {O}(10).$

**Fig. 8:**
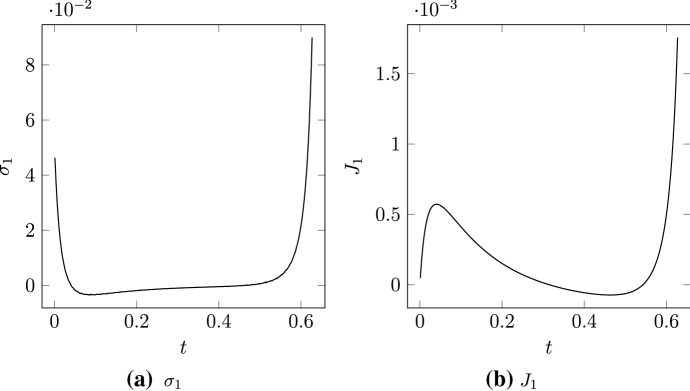
Calculation of $\sigma _1 \approx (\sigma - \sigma _0)/\varepsilon$ and $J_1 \approx (J - J_0)/\varepsilon$ in the numerical solution with $\varepsilon = 0.01.$ The values of $\sigma _0$ and $J_0$ were obtained using a solution with $\varepsilon = 1 \times 10^{-4}.$; **A** $\sigma _1.$; **B** $J_1.$

We also use the numerical solution with $\varepsilon = 0.01$ to validate the formula for $\sigma _1,$ the first-order correction to bulk stress. At each time step, we compute the stress $\sigma ,$ and compare with the stress in a simulation with $\varepsilon = 1 \times 10^{-4},$ which we consider to be $\sigma _0$ for rigid filaments. We then approximate the first-order correction as $\sigma _1 \approx (\sigma - \sigma _0)/\varepsilon ,$ and present results in Fig. 8A. For most values of $t,$ it holds that $\sigma _1 > 0.$ In particular, larger positive values of $\sigma _1$ occur close to $t = 0$ and $t = T,$ or $m = 0$ and $m = 1.$ Fig. 8A is surprising, because it suggests the introduction of filament bending generates stresses that are biased to expansion. Similarly, as Fig. 8B shows, the quantity $J_1(T) - J_1(0) > 0,$ also suggesting net expansive bias. Based on this, one might conclude that bending cannot facilitate microscopic-scale contraction. However, we have not yet accounted for the changes in filament geometry, and how they influence motor dynamics. Further simulations in Sect. 3.2.2 will reveal this more clearly, and confirm that bending does facilitate net microscopic-scale contraction.

#### Flexible filament solutions

**Fig. 9:**
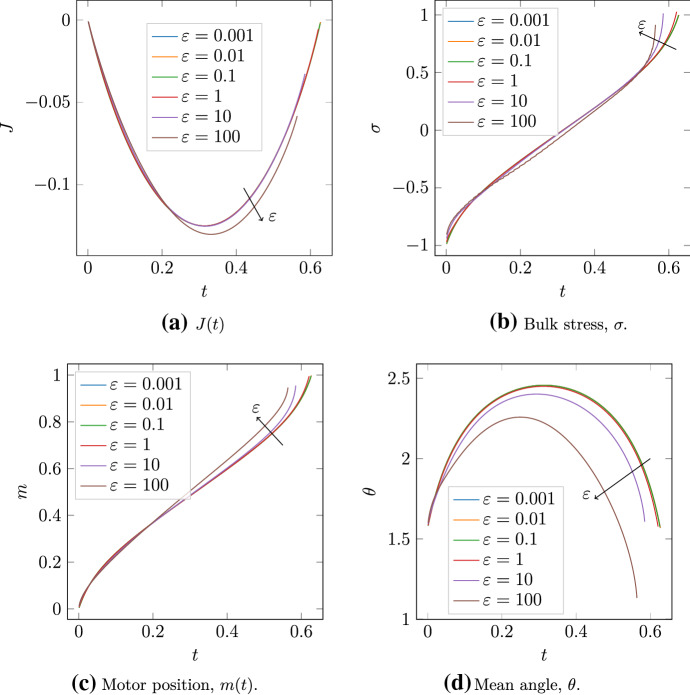
The effect of $\varepsilon$ on quantities in solutions of two symmetric filaments. Solutions are computed with $m(0) = 0,$ $\theta (0) = \pi /2,$ and proceed for all $t \in [0, T(\varepsilon )]$ such that $m(t) < 1.$ After this time $T,$ the motor reaches the plus-end and detaches. Results are plotted for six values of $\varepsilon ,$ and arrows indicate the direction of increasing $\varepsilon .$ **A** $J(t).$; **B** Bulk stress, $\sigma .$; **C** Motor position, $m.$ **D** Mean angle, $\theta .$

We now consider numerical solutions beyond the $\varepsilon \ll 1$ regime considered in the asymptotic analysis. These solutions are with the same conditions as Fig. 6, where the motor is initially at the minus-ends of two symmetric filaments. We then solve the model until the motor reaches the plus-ends. Results are presented in Fig. 9.

The quantity $J(t)$ measures the effect of $\varepsilon$ on net stress, and is plotted in Fig. 9A. For rigid filaments, we showed that $J(T) - J(0) = 0,$ indicating zero net stress as the motor moved from the minus to the plus-ends. Since $J(T)$ decreases as $\varepsilon$ increases, the introduction of filament bending facilitates bias to contraction. This contractile bias is despite the quantities $\sigma _1(T)$ and $J_1(T)$ being positive, as in Fig. 8. Indeed, Fig. 9B confirms that $\sigma _1 > 0,$ with stress increasing with $\varepsilon$ close to $t = 0$ and $t = T.$

Semi-flexible filaments facilitate net contraction because bending breaks the polarity-reversal symmetry, and the resulting geometry favours contraction. As Fig. 9C shows, with increasing $\varepsilon ,$ the myosin motor moves faster along the filaments and detaches earlier. The increase in motor speed is largest as the motor approaches the plus-ends, which Fig. 9B shows is associated with expansion. As the motor approaches the plus-ends, the semi-flexible filaments adopt a convex shape that brings them closer to parallel at their tips, as illustrated in Fig. 9D. This decreases the spring force through the motor, enabling it to move faster. Since the motor moves faster close to the plus-ends, the expansive component persists for shorter time than the contractile component. Consequently, the time-integrated stress $J(T) - J(0)$ decreases as $\varepsilon$ increases.

**Fig. 10:**
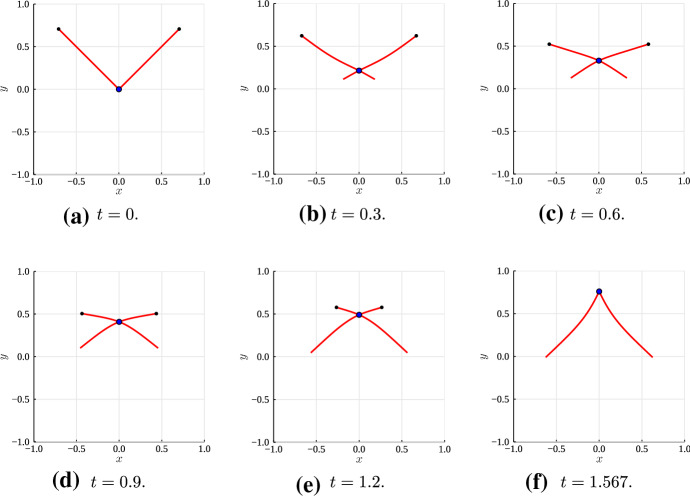
Numerical solution for the evolution of two flexible actin filaments (red solid curves) with $\varepsilon = 68.5$ and $V_m = 1.$ The black nodes indicate the filament plus ends, and the blue dot at the filament intersection represents the myosin motor. **A** $t = 0.$; **B** $t = 0.3.$; **C** $t = 0.6.$; **D** $t = 0.9.$; **E** $t = 1.2.$; **F** $t = 1.567.$

The results in Fig. 9 are relevant for *in vivo* actin filaments, for which the parameters (Kamasaki et al. 2007; Gittes et al. 1993; Thoresen et al. 2011; Reichl et al. 2008; Oelz et al. 2015) estimated in Tam et al. (2021) give $\varepsilon = 68.5.$ To further our analysis, we compute a numerical solution with $\varepsilon = 68.5$ and $V_m = 1,$ to investigate whether contraction persists after relaxing the assumption of infinite motor velocity. The evolution of these filaments is shown in Fig. 10.

**Fig. 11:**
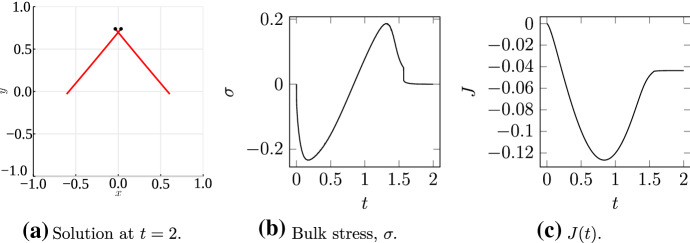
The final filament configuration, bulk stress, $\sigma ,$ and $J(t)$ for the flexible filament ($\varepsilon = 68.5$ and $V_m = 1$) solution in Fig. 10. (**A** Solution at $t = 2.$; **B** Bulk stress, $\sigma .$; **C** $J(t).$

Despite the slower motor speed, the evolution qualitatively follows the prediction from Fig. 7. Filaments are initially convex, then become concave, and adopt a convex shape again as the motor approaches the plus-ends. As Fig. 10F shows, the two filaments are curved when the motor reaches the plus-ends and detaches. To rule out the possibility that relaxation to straight configuration produces expansion that cancels out net contraction, we continued the simulation after motor detachment, until the filaments were again straight. We plot $\sigma (t)$ and $J(t)$ in Fig. 11. Although relaxation to the straight configuration (shown in Fig. 11A) generates a small amount of expansive stress, Fig. 11C shows $J(2) - J(0) < 0,$ suggesting net contraction. Thus, our proposed geometric mechanism for contraction remains relevant for realistic filament flexural rigidity and motor speed. Since actomyosin networks (for example those in the cortex) consist of many cross-linked two-filament assemblies, our mechanism provides a possible explanation for the microscopic origin of network-scale actomyosin contraction.

## Conclusion

Understanding the origins of actomyosin contraction is an open problem in cellular biophysics, with implications for cell movement and division. In this paper, we presented a detailed investigation of how a two-filament-motor system generates microscopic contraction if the filaments are flexible. We first derived a partial differential equation model, and described a method of computing in-plane stress. We then applied asymptotic analysis to a symmetric system with infinite free-moving motor velocity. The leading-order solution showed that two rigid filaments do not generate net stress if the motor traverses the entire length of the filaments. However, the introduction of filament bending enables the two-filament–motor structure to generate net contraction. This is because bending breaks the polarity-reversal symmetry of rigid filaments. The resulting geometric asymmetry draws the plus-ends closer to parallel as the motor approaches. This facilitates faster motor movement when motors are close to filament plus-ends, and inhibits production of expansive stress.

Our analysis confirms that the microscopic dynamics of symmetric filament pairs and motors can explain contraction. We expect that the same mechanism also favours contraction in non-symmetric filament–motor assemblies and that, consequently, macroscopic contraction in disordered networks could arise from the accumulation of multiple filament pairs, without the need for nonlinear amplification of contractile stress. Nevertheless, the question of how these results apply to disordered networks remains open. In disordered networks, filament pairs cross at arbitrary angle and position, and interact with an active background of other filaments, rather than the passive medium considered in this work. Tam et al. (2021) confirmed that disordered networks described by the biomechanical model for semi-flexible filaments and motors presented in this study do contract. Another potential approach modelling disordered network contraction is to derive a coarse-grained, continuum model based on the assumption of infinite filament length (Oelz 2014). This, and investigating how microscopic mechanics give rise to structures including stress fibres (Pellegrin and Mellor 2007) and the contractile ring (Kamasaki et al. 2007; Svitkina 2018), will be subjects of future work.

## Appendix A: mathematical model derivation

In this Appendix, we present a detailed derivation of the PDE model (2.10) -(2.12). We derive the system of force-balance PDEs using the variational principle. The functional for the structure consisting of a myosin motor attached to two semi-flexible actin filaments is

A1 $$\begin{aligned} \begin{aligned}&E[z_1,z_2,m_1,m_2] := \sum _{i=1}^2\int _0^{L_i} \left( \frac{\lambda _a}{2\Delta t}|z_i - z_i^n|^2 + \frac{\kappa _a}{2}|z_i''|^2 \right. \\&\quad \left. + \frac{1}{\delta _a} \left( \left|z_i'\right|- 1\right) ^2 \right) \,\text {d}s + \frac{1}{\delta _m}\left|z_1(m_1,t) - z_2(m_2,t)\right|^2 \\&\quad + \sum _{j=1}^2 \left( -F_s m_j + \frac{F_s}{V_m}\frac{\left( m_j - m_j^n\right) ^2}{2\Delta t} \right) , \end{aligned} \end{aligned}$$

where $z_i(s,t) = (x_i(s,t), y_i(s,t)),$ for $i = 1,2,$ are the filament shapes and positions, $m_1(t),$ and $m_2(t)$ are the motor relative positions. According to the variational principle underlying the derivation, the solution at the next time step is such that the functional derivative with respect to all degrees of freedom vanishes, that is

A2 $$\begin{aligned} \frac{\delta E}{\delta z_1} = 0, \quad \frac{\delta E}{\delta z_2} = 0, \quad \frac{\delta E}{\delta m_1} = 0, \quad \text { and } \quad \frac{\delta E}{\delta m_2} = 0. \end{aligned}$$

Evaluating each of (A2) gives rise to the variational equations

A3a $$\begin{aligned}&0 = \int _0^{L_1} \frac{\lambda _a}{\Delta t}\delta z_1 \cdot \left( z_1 - z_1^n\right) + \kappa _a z_1''\cdot \delta z_1'' + \frac{2}{\delta _a}(|z_1'|-1)\frac{ z_1'\cdot \delta z_1'}{\left|z_1'\right|} \,\text {d}s\nonumber \\&\qquad + \frac{2}{\delta _m}\left( z_1(m_1, t)-z_2(m_1, t)\right) \cdot \delta z_1\left( m_1,t\right) , \end{aligned}$$

A3b $$\begin{aligned}&0 = \int _0^{L_2} \frac{\lambda _a}{\Delta t}\delta z_2 \cdot \left( z_2 - z_2^n\right) + \kappa _a z_2''\cdot \delta z_2'' + \frac{2}{\delta _a}(|z_2'|-1)\frac{ z_2'\cdot \delta z_2'}{\left|z_2'\right|} \,\text {d}s\nonumber \\&\qquad - \frac{2}{\delta _m}\left( z_1(m_1, t)-z_2(m_1, t)\right) \cdot \delta z_2\left( m_2,t\right) , \end{aligned}$$

A3c $$\begin{aligned}&0 = \delta m_1\left[ -F_s + \frac{F_s}{V_m}\frac{\left( m_1-m_1^n\right) }{\Delta t} \right. \nonumber \\&\qquad \left. + \frac{2}{\delta _m}\left( z_1(m_1, t)-z_2(m_1, t)\right) \cdot z_1'\left( m_1, t\right) \right] , \end{aligned}$$

A3d $$\begin{aligned}&0 = \delta m_2\left[ -F_s + \frac{F_s}{V_m}\frac{\left( m_2-m_2^n\right) }{\Delta t} \right. \nonumber \\&\qquad \left. - \frac{2}{\delta _m}\left( z_1(m_1, t)-z_2(m_1, t)\right) \cdot z_2'\left( m_2,t\right) \right] . \end{aligned}$$

For the motor evolution equations (A3c) and (A3d), we can immediately apply the continuum limit $\Delta t \rightarrow 0,$ for which $\dot{m}_i = (m_i-m_i^n)/\Delta t$ for $i = 1,2.$ In addition, we consider the limit $\delta _m \rightarrow 0$ and replace the forces due to motor stretching by the force $\mu$ which represents the Lagrange multiplier for the constraint

A4 $$\begin{aligned} z(m_1,t) = z(m_2,t). \end{aligned}$$

This yields the ordinary differential equations

A5a $$\begin{aligned} \frac{\text {d}m_1}{\text {d}t}&= V_m\left[ 1 - \frac{\mu }{F_s}\frac{z_1-z_2}{\Vert z_1-z_2\Vert }\cdot z_1'(m_1,t)\right] , \end{aligned}$$

A5b $$\begin{aligned} \frac{\text {d}m_2}{\text {d}t}&= V_m\left[ 1 + \frac{\mu }{F_s}\frac{z_1-z_2}{\Vert z_1-z_2\Vert }\cdot z_2'(m_2,t)\right] , \end{aligned}$$

which are force-balance equations for the myosin motor positions $m_1(t)$ and $m_2(t).$ The equations (A5) represent that unloaded motors (for which $\mu = 0$) move at the free-moving velocity, $V_m.$ As the motor moves, it is exposed to stretching forces, with magnitude given by the Lagrange multiplier $\mu .$ The term involving the dot product is the projection of this force onto the direction of motor movement along the $i$-th filament. Assuming a linear force–velocity relation, the ratio of the term involving $\mu$ and the dot product to the stall force, $F_s,$ determines the reduction of motor speed due to stretching forces.

For the filament equations (A3a) and (A3b), in addition to $\delta _m \rightarrow 0$, we also let $\delta _a \rightarrow 0$ enforcing the constraints

A6 $$\begin{aligned} |z_i'|= 1, \quad i=1,2. \end{aligned}$$

We write the limits of $2(|z_i'|-1)/\delta _a$ as $\lambda _i$ and apply integration by parts to remove derivatives of $\delta z_i$ from under the integral sign. This yields

A7 $$\begin{aligned} \begin{aligned} \int \kappa _a z_i''\cdot \delta z_i'' + \lambda _i z_i' \cdot \delta z_i' \,\text {d}s&= \int \delta z_i\cdot \left[ \kappa _az_i'''' - \left( \lambda _iz_i'\right) '\right] \,\text {d}s \\&\quad + \delta z_i\cdot \left( \lambda _iz_i' - \kappa _az_i'''\right) + \kappa _a\delta z_1'\cdot z_i''. \end{aligned} \end{aligned}$$

We then rewrite equations (A3a) and (A3b) as

A8a $$\begin{aligned}&\int _{0}^{L_1} \delta z_1 \cdot \left[ \frac{\lambda _a}{\Delta t}\left( z_1 - z_1^n\right) + \kappa _az_1'''' - \left( \lambda _1z_1'\right) '\right. \nonumber \\&\quad \left. + \mu \frac{z_1-z_2}{\Vert z_1-z_2\Vert }\delta \left( s-m_1\right) \right] \,\text {d}s + \left[ \delta z_1\cdot \left( \lambda _1z_1' - \kappa _az_1'''\right) + \kappa _a\delta z_1'\cdot z_1''\right] _{0}^{L_1}, \end{aligned}$$

A8b $$\begin{aligned}&\int _{0}^{L_2} \delta z_2 \cdot \left[ \frac{\lambda _a}{\Delta t}\left( z_2 - z_2^n\right) + \kappa _az_2'''' - \left( \lambda _2z_2'\right) ' \right. \nonumber \\&\quad \left. - \mu \frac{z_1-z_2}{\Vert z_1-z_2\Vert }\delta \left( s-m_2\right) \right] \,\text {d}s + \left[ \delta z_2\cdot \left( \lambda _2z_2' - \kappa _az_2'''\right) + \kappa _a\delta z_2'\cdot z_2''\right] _{0}^{L_2}, \end{aligned}$$

where $\delta$ is the Dirac delta function. The equations (A8) enable us to derive the continuum governing equations and boundary conditions. First, we require the filaments to have zero curvature at their tips,

A9 $$\begin{aligned} z_i'' = 0 \quad \text { at } \quad s = 0, L_i. \end{aligned}$$

The remaining boundary terms in (A8) then give rise to the conditions

A10 $$\begin{aligned} \lambda _iz_i' - \kappa _az_i''' = 0 \quad \text { at } \quad s = 0, L_i, \end{aligned}$$

which specifies that the boundary values vanish at $s = 0, L_i.$ Next, we apply the fundamental lemma of the calculus of variations to the remaining integrals. In the continuum limit $\Delta t \rightarrow 0$ for which $(z_i - z_i^n)/\Delta t = \dot{z}_i,$ we obtain

A11a

A11b

The differential equations (A11) and (A5), and conditions (A9) and (A10) as well as the constraints (A4) and (A6) then define a system of force-balance equations and boundary conditions that govern the evolution of two inextensible, semi-flexible filaments connected to an inextensible motor. This is the dimensional PDE model (2.10)-(2.12) given in the main text.

## Appendix B: Asymptotic analysis

In this Appendix, we present the asymptotic analysis of Sect. 3.1 in more detail. We first outline the method used to solve the leading-order problem (3.6), and subsequently consider the $\mathcal {O}(\varepsilon )$ problem (3.14) for the first-order corrections.

### Leading-order problem

We commence the analysis by considering the ansatz for rigid filaments. Taking the time derivative of (3.3) gives  and taking four spatial derivatives of (3.7) yields $z_1^{(5)}=h^{(5)} z_0'^\perp .$ Next, we can differentiate the governing equation of $\mathcal {O}(1)$ (3.6a) once with respect to $s,$ and substitute the two above expressions to obtain

B1 $$\begin{aligned} -z_0'^\perp \frac{\dot{\theta }}{2} + h^{(5)}z_0'^\perp -\lambda _0'' z_0' + \mu _0 \begin{pmatrix} 1 \\ 0 \end{pmatrix} \delta '(s-m_0) = 0. \end{aligned}$$

We can now multiply the boundary condition (3.6c) by $z_0',$ and use the property $z_1''' \cdot z_0' = 0,$ which follows from the orthogonality condition (3.6e), to obtain $\lambda _0(0) = \lambda _0(1) = 0.$ Finally, multiplying (B1) by $z_0'$ implies that

B2 $$\begin{aligned} \lambda _0=\mu _0 \begin{pmatrix} 1 \\ 0 \end{pmatrix} \cdot z_0' \left( H(s-m_0)-s\right) =H(s-m_0)-s, \end{aligned}$$

where $H(s)$ denotes the Heaviside step function. The Lagrange multiplier $\mu _0 = 1/\sin (\theta /2),$ by rearranging (3.6b). The leading-order bulk stress is then

B3 $$\begin{aligned} \sigma _0 = -2 \int _0^1 \lambda _0 \,\text {d}s =-2 \mu _0 \sin \left( \frac{\theta }{2}\right) \left( \frac{1}{2}-m_0\right) = -2\left( \frac{1}{2}-m_0\right) = 2 \nu _0, \end{aligned}$$

where $\nu _0 = m_0 - 1/2.$ This completes the derivation of the quantities listed in (3.8).

We now derive the ordinary differential equations (3.9) for $y_0,$ $\theta ,$ and $\nu _0,$ the three degrees of freedom that govern the leading-order filament position, $z_0.$ On taking the time derivative and variation, the ansatz (3.5) implies that

B4a

B4b

Integrating (3.6a) against $\delta z_0$ then yields

B5

Substituting (B4) into (B5) then gives

B6 $$\begin{aligned} 0&= \int _0^1 \left[ \begin{pmatrix} 0 \\ \dot{y}_0 \end{pmatrix} - z_0' \dot{m}_0 - z_0'^\perp (s- m_0) \frac{\dot{\theta }}{2} \right] \cdot \left[ \begin{pmatrix} 0 \\ \delta y_0 \end{pmatrix} \right. \nonumber \\&\left. \qquad - z_0' \delta m_0 - z_0'^\perp (s- m_0) \frac{\delta \theta }{2} \right] \,\text {d}s - \delta m_0\nonumber \\&= \begin{pmatrix} 0 \\ \delta y_0 \end{pmatrix} \cdot \int _0^1 \left[ \begin{pmatrix} 0 \\ \dot{y}_0 \end{pmatrix} - z_0' \dot{m}_0 - z_0'^\perp (s- m_0) \frac{\dot{\theta }}{2} \right] \,\text {d}s \nonumber \\&\qquad - \delta m_0\left[ \begin{pmatrix} 0 \\ \dot{y}_0 \end{pmatrix} \cdot z_0' - \dot{m}_0 \right] \nonumber \\&\qquad - \int _0^1 \left[ z_0'^\perp \cdot \begin{pmatrix} 0 \\ \dot{y}_0 \end{pmatrix} - (s - m_0)\frac{\dot{\theta }}{2} \right] \cdot (s- m_0) \frac{\delta \theta }{2} \,\text {d}s - \delta m_0 \nonumber \\&= \delta y_0 \int _0^1 \left( \dot{y}_0 - \cos (\theta /2) \dot{m}_0 - \sin (\theta /2) (s - m_0) \frac{\dot{\theta }}{2} \right) \,\text {d}s\nonumber \\&\qquad - \delta m_0 \left( \dot{y}_0 \cos (\theta /2) - \dot{m}_0 \right) \nonumber \\&\qquad - \int _0^1 \left( \dot{y}_0 \sin (\theta /2) - (s - m_0) \frac{\dot{\theta }}{2} \right) \cdot (s - m_0) \frac{\delta \theta }{2} \,\text {d}s - \delta m_0 \nonumber \\&= \delta y_0 \left( \dot{y}_0 - \cos (\theta /2) \dot{m}_0 - \sin (\theta /2) (1/2- m_0) \frac{\dot{\theta }}{2} \right) \nonumber \\&\qquad - \delta m_0 \left( 1+\dot{y}_0 \cos (\theta /2) - \dot{m}_0 \right) \nonumber \\&\qquad - \left( \dot{y}_0 \sin (\theta /2) (1/2 - m_0) - (1/3 - m_0+m_0^2) \frac{\dot{\theta }}{2} \right) \frac{\delta \theta }{2}. \end{aligned}$$

Collecting the coefficients of $\delta y_0,$ $\delta \theta$ and $\delta m_0,$ we obtain the system of differential equations (writing $\nu _0=m_0-1/2$)

B7a $$\begin{aligned} \dot{y}_0&=\left( 12\nu _0^2+1\right) \cot \left( \frac{\theta }{2}\right) \csc \left( \frac{\theta }{2}\right) , \end{aligned}$$

B7b $$\begin{aligned} \dot{\theta }&= -24\nu _0 \cot \left( \frac{\theta }{2}\right) , \end{aligned}$$

B7c $$\begin{aligned} \dot{\nu }_0&= \csc ^2\left( \frac{\theta }{2}\right) \left( 1+6 \nu _0^2 ( \cos (\theta )+1)\right) , \end{aligned}$$

where $\csc (\phi )=1/\sin (\phi ).$ The equations (B7b) and (B7c) for $\theta$ and $\nu _0$ are also independent of $y_0,$ suggesting that the solution is invariant to vertical translations. Furthermore, the trigonometric functions can be eliminated by writing $S = \sin ^2(\theta /2),$ which yields

B8a $$\begin{aligned} \frac{\text {d}S}{\text {d}t}&= -24 \nu _0 \left( 1-S \right) , \end{aligned}$$

B8b $$\begin{aligned} S\frac{\text {d}\nu _0}{\text {d}t}&= 1+12 \nu _0^2 (1-S). \end{aligned}$$

This completes the derivation of equation (3.9).

### First-order problem

To obtain the higher-order corrections, we first use the leading-order equation (B1) and the ansatz (3.7) to solve for $h,$ the curvature of $z_1.$ Multiplying (B1) by $z_0'^\perp$ and using $z_1''' \cdot z_0' = 0$ (which follows from the orthogonality condition (3.6e)), we obtain $h'''(0)=h'''(1)=0,$ and subsequently

B9 $$\begin{aligned} -\frac{\dot{\theta }}{2} + h''''' - \mu _0 \cos \left( \frac{\theta }{2}\right) \delta '(s-m_0) = 0. \end{aligned}$$

The boundary conditions $h'''(0)=0=h'''(1)$ imply that

B10 $$\begin{aligned} h''' = \frac{\dot{\theta }}{2}\frac{s(s-1)}{2} + \mu _0 \cos \left( \frac{\theta }{2}\right) \left( H(s-m_0)-s \right) \end{aligned}$$

and furthermore (since $z_1''(0) = 0=z_1''(1)$), substituting for $\dot{\theta }$ and $\mu _0,$

B11 $$\begin{aligned} h''=-\cot \left( \frac{\theta }{2}\right) \left[ (m_0-s) H(s-m_0)+s^2 (m_0 (2 s-3)-s+2)\right] , \end{aligned}$$

which is an expression for filament curvature, $h''(s,t).$

We now obtain the perturbation solution for the bulk stress, $\sigma _1,$ which requires knowledge of the quantities $\mu _1$ and $\lambda _1.$ First, we integrate (B11) once with respect to $s.$ This introduces another constant of integration, denoted $A(t),$ which might be time-dependent and cannot be determined from boundary data. Consequently, we write

B12 $$\begin{aligned} h'(s,t) = \tilde{h}'(s,t) + A(t), \end{aligned}$$

where

B13 $$\begin{aligned} \tilde{h}'(s, t) = \frac{\dot{\theta }}{2} \frac{s^3}{12} \left( \frac{s}{2}-1\right) + \mu _0\cos \left( \frac{\theta }{2}\right) \left( \frac{1}{2}(m - s)^2 H(s-m)- \frac{s^3}{6 }\right) . \end{aligned}$$

Collecting the coefficients of $\varepsilon$ in the governing equations with asymptotic expansions, we obtain the $\mathcal {O}(\varepsilon )$ problem (3.14). On taking a derivative of (3.14a) and substituting $z_1' = h'(s,t)z_0'^\perp =(A(t)+\tilde{h}'(s,t)) z_0'^\perp ,$ we obtain

B14

Expanding the inextensibility constraint (3.14f) implies that $z_0' \cdot z_2^{(5)}= -(|z_1'|^2)^{(4)}/2.$ Multiplying (B14) by $z_0',$ we obtain

B15 $$\begin{aligned} - \frac{1}{2} (|z_1'|^2)^{(4)}-\lambda _1'' + \begin{pmatrix} 1 \\ 0 \end{pmatrix}\cdot z_0' \left[ \mu _1 \delta '(s-m_0)-\mu _0\delta ''(s-m_0)m_1 \right] = 0. \end{aligned}$$

We can integrate (B15) twice and apply the boundary conditions (3.14c) to determine the constants of integration. This yields

B16 $$\begin{aligned} \frac{1}{2} (|z_1'|^2)''+\lambda _1 = \begin{pmatrix} 1 \\ 0 \end{pmatrix} \cdot z_0' \left[ \mu _1 \left( H(s-m_0)- \frac{s}{L} \right) -\mu _0\delta (s-m_0)m_1 \right] , \end{aligned}$$

which we can rearrange to obtain $\lambda _1.$ To eliminate $\mu _1$ from (B16), we use (3.14b) to infer an expression for $\mu _1.$ Substituting the ansatzes (3.5) and (3.7) for $z_0$ and $z_1$ respectively, we obtain

$$\begin{aligned} 0&= -\begin{pmatrix} 1 \\ 0 \end{pmatrix} \cdot \left( \mu _1 z_0'+ \mu _0 h'(s,t) z_0'^\perp (m_0,t) \right) \\&=-\left[ \sin \left( \frac{\theta }{2} \right) \mu _1 - \cos \left( \frac{\theta }{2} \right) \mu _0 h'(m_0,t) \right] , \end{aligned}$$

and therefore

B17 $$\begin{aligned} \mu _1=\mu _0 h'(m_0, t) \frac{\cos \left( \theta /2\right) }{\sin \left( \theta /2\right) } = \mu _0 (A+\tilde{h}'(m_0,t)) \cot \left( \frac{\theta }{2}\right) . \end{aligned}$$

Substituting (B17) into (B16), we obtain

B18 $$\begin{aligned} \lambda _1&= \begin{pmatrix} 1 \\ 0 \end{pmatrix} \cdot z_0' \left[ \left( \mu _0 (A+\tilde{h}'(m_0, t)) \cot \left( \frac{\theta }{2} \right) \right) \left( H(s-m_0)- s \right) \right. \nonumber \\&\qquad \left. -\mu _0 \delta (s-m_0)m_1 \right] - \frac{1}{2}\left( |z_1'|^2\right) '' \nonumber \\&= \left( A + \tilde{h}'(m_0, t) \right) \cot \left( \frac{\theta }{2} \right) \left( H(s-m_0)- s \right) - \delta (s-m_0)m_1 \nonumber \\&\qquad - (z_1' \cdot z_1'')'. \end{aligned}$$

Using the simplified expression (B18) for $\lambda _1,$ we can write the first-order perturbation of the bulk stress (3.12), given by

B19 $$\begin{aligned} \sigma _1&= -2\int _{0}^{1} \left( \left|z_1''\right|^2 + \lambda _1 \right) \,\text {d}s\nonumber \\&= -2\int _{0}^{1} \left|h''\right|^2 \,\text {d}s -2 \left( A+ \tilde{h}'(m_0, t) \right) \cot \left( \frac{\theta }{2} \right) \left( \frac{1}{2}-m_0 \right) +2m_1, \end{aligned}$$

where $$\tilde{h}'(m_0, t)$$ is

B20 $$\begin{aligned} \tilde{h}'(m_0, t)&=\frac{\dot{\theta }}{2} \frac{m_0^3}{12} \left( \frac{m_0}{2}-1\right) + \cot \left( \frac{\theta }{2}\right) \left( - \frac{m_0^3}{6 }\right) \nonumber \\&=\frac{1}{2} \left( -12 (2 m_0-1) \cot \left( \frac{\theta }{2}\right) \right) \frac{m_0^3}{12} \left( \frac{m_0}{2}-1\right) \nonumber \\&\qquad + \cot \left( \frac{\theta }{2}\right) \left( - \frac{m_0^3}{6 }\right) \nonumber \\&=\cot \left( \frac{\theta }{2}\right) \left( - \frac{m_0^3}{12}\right) \left[ \left( 12 m_0-6\right) \left( \frac{m_0}{2}-1\right) + 2\right] \nonumber \\&=-\frac{1}{12} m_0^3 \left( 6 m_0^2-15 m_0+8\right) \cot \left( \frac{\theta }{2}\right) . \end{aligned}$$

To solve for the first-order correction to the filament positions, $z_1,$ we require an initial condition, here denoted $z_{I,1}(s) = z_1(s, 0).$ To determine the asymptotic expansion of the initial condition (2.29c), we return to the force-balance equations (2.1), and its equivalent time-discrete minimisation problem for the functional (2.2). In this expression, the drag component (2.3) dominates when $\Delta t$ is small. Therefore, we determine the leading order term in the asymptotic expansion of the initial condition $z_I=z_{I,0}+\varepsilon z_{I,0} + \dots$ as the best approximation of $z_I$ in $L^2$ among the straight fibres (3.5), that is

B21 $$\begin{aligned} z_{I,0} = \begin{pmatrix} 0 \\ y_{I,0} \end{pmatrix} + \begin{pmatrix} \sin (\theta _I/2) \\ \cos (\theta _I/2) \end{pmatrix} \left( s - m_{I,0}\right) , \end{aligned}$$

where

B22 $$\begin{aligned} (m_{I,0}, y_{I,0}, \theta _I) = \mathrm{argmin}_{\bar{m}, \bar{y}, \bar{\theta }} \int _0^1 \left( z_{I,0}-z_I \right) ^2 \,\text {d}s. \end{aligned}$$

Since we focus on pairs of initially straight fibres in this study, we set $z_I = z_{I,0}.$

A similar approach is available to determine $z_{I,1}.$ Using the ansatz (3.7), we have

B23 $$\begin{aligned} \begin{aligned} z_{I,1}&= \begin{pmatrix} 0 \\ y_{I,1}(t) \end{pmatrix} \\&\qquad - z_{I,0}'(t) m_{I,1}+ z_{I,0}'^\perp \left( A_I(s-m_{I,0})+\int _{m_{I,0}}^s \tilde{h}'(t,s) \,\text {d}s \right) , \end{aligned} \end{aligned}$$

where

B24 $$\begin{aligned} (m_{I,1}, y_{I,1}, A_I) = \mathrm{argmin}_{\bar{m}, \bar{y}, \bar{\theta }} \int _0^1 \left( z_{I,0} + \varepsilon z_{I,1}-z_I \right) ^2 \,\text {d}s. \end{aligned}$$

In the case where $z_{I,0}=z_I$ the term $z_{I,1}$ is minimal in $L^2.$ Then, the degrees of freedom $m_{I,1},$ $y_{I,1},$ and $A_I$ can be computed using

B25 $$\begin{aligned} 0= \int _0^1 z_{I,1} \cdot \delta z_{I,1} \,\text {d}s, \end{aligned}$$

where

B26 $$\begin{aligned} \delta z_{I,1}= \begin{pmatrix} 0 \\ \delta y_1 \end{pmatrix} - z_{I,0}'\delta m_1 + z_{I,0}'^\perp (s-m_{I,0})\delta A. \end{aligned}$$

When we set $\delta y_1=y_0,$ $\delta m_1=\int _0^1 (m_0-s) \,\text {d}s,$ and $\delta A=0,$ we find that

B27 $$\begin{aligned} 0=\int _0^1 z_{I,1} \cdot \left[ \begin{pmatrix} 0 \\ y_{I,0} \end{pmatrix} + z_{I,0}' (s- m_{I,0}) \right] = \int _0^1 z_{I,1} \cdot z_{I,0} \,\text {d}s. \end{aligned}$$

It therefore holds that $J_1(0)=0.$

To complete the derivation, we use the ansatz (3.7) with degrees of freedom $A(t),$ $y_1(t),$ and $m_1(t).$ Its variation and time-derivative are given by

B28a

B28b

where $\tilde{h}'(m_0, t)$ is given in (B20). A system of differential equations for $m_1,$ $A$ and $y_1$ can then be found integrating (3.14a) against $\delta z_1.$ Using computer algebra, we obtained the system

B29a $$\begin{aligned} \frac{\text {d}y_1}{\text {d}t}&= \frac{1}{960} \csc ^4\left( \frac{\theta }{2}\right) \left[ 960 A \sin \left( \frac{\theta }{2}\right) \left( 6 \nu _0^2 \cos (\theta )+6 \nu _0^2+1\right) \right. \nonumber \\&\qquad \left. + \cos \left( \frac{\theta }{2}\right) \left( -8928 \nu _0^7+9600 \nu _0^5-720 \nu _0^4+490 \nu _0^3+2940 \nu _0^2\right. \right. \nonumber \\&\qquad \left. \left. +3 \nu _0 (960 m_1-17)+240\right) \right. \nonumber \\&\qquad \left. - 3 \cos \left( \frac{3 \theta }{2}\right) \left( 864 \nu _0^7-960 \nu _0^5-240 \nu _0^4+30 \nu _0^3+980 \nu _0^2\right. \right. \nonumber \\&\qquad \left. \left. +3 \nu _0+960 \nu _0 m_1+80\right) \right] ,\end{aligned}$$

B29b $$\begin{aligned} \frac{\text {d}m_1}{\text {d}t}&= -\frac{1}{960} \csc ^4\left( \frac{\theta }{2}\right) \left[ -5760 A \nu _0^2 \sin (\theta )-480 A \sin (\theta )\right. \nonumber \\&\qquad \left. + 10 \left( 576 \nu _0^7-624 \nu _0^5-20 \nu _0^3+3 \nu _0+48\right) \cos (\theta )\right. \nonumber \\&\qquad \left. +4608 \nu _0^7-5040 \nu _0^5-200 \nu _0^3- 1440 \nu _0^2+21 \nu _0 \right. \nonumber \\&\qquad \left. + 3 \nu _0 \cos (2 \theta ) \left( 384 \nu _0^6-400 \nu _0^4+480 \nu _0+480 m_1 +3\right) \right. \nonumber \\&\qquad \left. -1440 \nu _0 m_1-480\right] , \end{aligned}$$

B29c $$\begin{aligned} \frac{\text {d}A}{\text {d}t}&= -\frac{1}{160} \cot \left( \frac{\theta }{2}\right) \csc ^2\left( \frac{\theta }{2}\right) \left[ 576 \nu _0^6-880 \nu _0^4+720 \nu _0^3-300 \nu _0^2\right. \nonumber \\&\qquad \left. -900 \nu _0-15+ 4 \left( 96 \nu _0^5-120 \nu _0^3+60 \nu _0^2+245\right) \nu _0 \cos (\theta )\right] . \end{aligned}$$

These ODEs govern the solution for $z_1,$ the first-order correction to the filament shape.
